# Facile and efficient 3-chlorophenol sensor development based on photolumenescent core-shell CdSe/ZnS quantum dots

**DOI:** 10.1038/s41598-019-57091-6

**Published:** 2020-01-17

**Authors:** Mohammed M. Rahman, Mohammad Rezaul Karim, M. M. Alam, M. Badruz Zaman, Nabeel Alharthi, Hamad Alharbi, Abdullah M. Asiri

**Affiliations:** 1 Center of Excellence for Advanced Materials Research (CEAMR) & Chemistry Department, Faculty of Science, King Abdulaziz University, Jeddah, 21589 Saudi Arabia; 2 Center of Excellence for Research in Engineering Materials (CEREM), Deanship of Scientific Research (DSR), King Saud University, Riyadh 11421 & K.A.CARE Energy Research and Innovation Center, Riyadh, 11451 Saudi Arabia; 3 Department of Chemical Engineering and Polymer Science, Shahjalal University of Science and Technology, Sylhet, 3100 Bangladesh; 4 Quality Engineering Test Establishment, Department of National Defence, Gatineau, QC J8X 1C6 Canada; 5 Mechanical Engineering Department, College of Engineering, King Saud University, Riyadh, 11421 Saudi Arabia

**Keywords:** Quantum dots, Quantum dots

## Abstract

Quantum dots (QDs) are semiconducting inorganic nanoparticles, tiny molecules of 2–10 nm sizes to strength the quantum confinements of electrons. The QDs are good enough to emit light onto electrons for exciting and returning to the ground state. Here, CdSe/ZnS core/shell QDs have been prepared and applied for electrochemical sensor development in this approach. Flat glassy carbon electrode (GCE) was coated with CdSe/ZnS QDs as very thin uniform layer to result of the selective and efficient sensor of 3-CP (3-chlorophenol). The significant analytical parameters were calculated from the calibration plot such as sensitivity (3.6392 µA µM^−1^ cm^−2^) and detection limit (26.09 ± 1.30 pM) with CdSe/ZnS/GCE sensor probe by electrochemical approach. The calibration curve was fitted with the regression co-efficient r^2^ = 0.9906 in the range of 0.1 nM ∼ 0.1 mM concentration, which denoted as linear dynamic range (LDR). Besides these, it was performed the reproducibility in short response time and successfully validated the fabricated sensor for 3-CP in the real environmental and extracted samples. It is introduced as a noble route to detect the environmental phenolic contaminants using CdSe/ZnS QDs modified sensor by electrochemical method for the safety of healthcare and environmental fields at broad scales.

## Introduction

Semiconductor quantum dots (QDs), in particular those of cadmium chalcogenides (CdS, CdSe, CdTe), have received steadily growing attention due to their high potential for various applications, such as sensing^1,2^, biological labeling^3–12^, light-emitting devices (LED)^13–17^, lasers^18,19^, and solar cells^20–22^. Recently, the number of interest has been developed in the area of electrochemical sensors and biosensors in which CdSe are employed for the determination of clinical markers^23^ and food^24^ and environmental pollutants^25,26^. CdSe have been using as electrochemical signal tracers to exploit the oxidation potentials of the metal ions forming them. This can also be attributed to some of the characteristics of CdSe, such as a large surface area–to–volume ratio, good interfacial properties with high surface reaction activity, high electron-transfer efficiency, excellent biocompatibility, and feasibility for surface modification^27–30^. Moreover, metallic QDs also constitute a feasible option for electrochemical multiplexed assays due to their different stripping peak potentials^31,32^. This is particularly true for the development of efficient, reliable, rapid, and cost-affordable analytical procedures for the determination of anthropogenic and natural substances of either organic or mineral nature that have toxic, persistent, and bio-accumulative properties.

Due to the prevalent pollution of ground and surface water by chloro-organic chemicals such as chlorophenol derivatives, the public concern has been growing over the several few decades. Because, the chlorophenols (including 3-CP) have high toxicity and able to produce mutagenic, estrogenic and carcinogenic effects in human beings^33–36^ and even unsafe for the environment and ecological systems. Thus, it is responsible for stimulating the central nervous and respiratory systems in case of lower doses exposure, but higher level can be produced cancer^37–39^. As a result, the USA Environmental Protection Agency (EPA) has considered the chlorophenol as priority hazardous toxic chemicals^40^. The sources of chlorophenols in environment are produced by man-made and industrial activities such as chlorinated bleaching of pulp, hydrolysis of chlorinated herbicides and oil refining. Besides this, the chlorophenol is used as disinfectant in municipality’s water, agro-fungicides and chlorinated pesticides. All the results are responsible for contamination of ground and surface water^41–43^. Therefore, a sensitive and comfort system is necessary to analyze the environmental (both ground and surface) and portable water. The existing analytical methods such as fluorescence and Raman spectrophotometry^44–46^, photo-electrochemical methods^47–50^, chromatography^51,52^, chemi-luminescence^53–55^, and electrochemical methods^29,56–60^ are used widely to detect the chlorophenol in aqueous medium. Among the chlorophenol detection methods, the electrochemical method has better advantages and the electrochemical method based on current versus potential is becoming more popular to detect directly the toxic chemicals which are environmental unsafe and carcinogenic^61–64^.

The above methods of this proposed detection are with narrow linear dynamic ranges as well as the higher detection limits, although they are expensive due to maintain sophisticated equipment, time consuming tests and needs skilled operators. Additionally, systematic errors are found during the analysis as for the essential sample pre-treatment and required longer preparation time. Advancement of portable, cheaper and quick responding devices for detecting metal ions are remarkable over the last few decades. Many industries are using chemo-sensors to detect metal ions in water as the capability for both on-line and field monitoring^65^. These industries are needed the accurate sensor with higher sensitivity, quick response time, superior signal-to-noise ratio, high selectivity towards the target analyte, mathematical relationship of signal yield to the amount of analyte, and long-term stability. There are various sensors have been introduced including electrochemical, optical, colorimetric and fluorescent sensors and so on. To the best of our knowledge, this is the first approach for the development of selective and efficient chemical sensor with CdSe/ZnS core/shell type QDs by using electrochemical method.

In this study, CdSe/ZnS QD was implemented onto the flat surface of GCE with the conducting 5% nafion as a uniform thin layer to fabricate the proposed 3-CP electrochemical sensor at room conditions. It was utilized to detect the 3-CP analyte by electrochemical method in phosphate buffer phase. Analytical performances of the proposed electrochemical sensor such as sensing capability, response time, detection limit and linear dynamic range were calculated very carefully. Beside this, the 3-CP sensor was anticipated to analyze the real environmental samples successfully. Therefore, this novel research approach is to develop an efficient detection of target 3-CP in phosphate buffer phase might be a milestone in field of environmental and health care fields in a broad scale.

## Experimental Section

### Materials and method

Cadmium oxide (Aldrich, 99.99%), sulphur (99.9% powder, Anachemia Chemicals Ltd.), selenium (Aldrich), tributylphosphine oxide (Aldrich), octadecylamine (Aldrich, ODA), zinc oxide (Alfa Aesar), ODE (1-octadecene, Aldrich, 90%), oleic acid (Aldrich), tri-n-octylphosphine (Aldrich), tributylphosphine (Aldrich, TBP), stearic acid (Aldrich, SA) and oleic acid (Aldrich, OA). To complete this research, the toxic chemicals such as 3,4-diaminotoluene, 3-chlorophenol, 3-methoxyphenol, 4-aminophenol, 4-nitrophenyhydrazine, acetonitrile, benzylchloride, chlorobenzene, p-nitrophenol and toluene were obtained from the sigma Aldrich supplier and used directly without any refinement. To execute the electrochemical investigation, a Keithly Electrometer was used to analyze into electrochemical cell by electrochemical investigation.

### Synthesis of quantum dots

**Figure 1:**
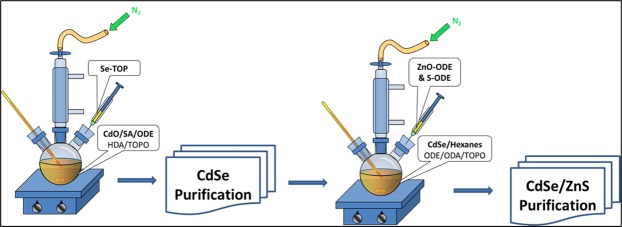
The schematic synthesis route of CdSe/ZnS core-shell QDs.

Tri-n-octylphosphine (TOP), tributylphosphine oxide (TOPO) or TOPO/stearic acid capped CdSe QDs were prepared by using the previous method, with modification^66,67^. In a reaction mixture, 2.5 mmol of cadmium oxide, 7.0 mmol of SA with 50.0 mL of ODE in a 250 mL three-neck flask was heated about 200~290 °C to get a clear and colorless solution. Cooling the solution around 100~120 °C, it was added HDA (18.0 g) & TOPO (10 g) in the solution. The mixture was reheated to 280 °C under the nitrogen supply. At 280 °C, Se-TOP (2.1 mL) was added quickly in the above solution. It was controlled the reaction temperature about 200~290 °C for 10~15 min and allowed it to cool down at normal temperature. The resultant mixture was dissolved in hexanes or toluene and transferred into a separating funnel; about equal volume of MeOH was added and extracted vigorously for minimum 30 min. Afterwards, the funnel was kept overnight for the clear occurrence of two layers: the nanocrystals remained in the upper layer, namely the hexanes/ODE layer or toluene/ODE layer. Usually, three times purification was performed to obtain a clear and transparent layer of hexane/toluene, which is used to measure the concentration of nanocrystal using UV absorption measurement^68^. ZnS shell with a few monolayers was grown on the purified CdSe QDs, by the successive ion layer adhesion and reaction method^69^. Based on the CdSe nanocrystal concentration obtained, we calculated the zinc and sulphur precursors needed for the ZnS coating, the reaction temperature was controlled from 200 to 240 °C. The CdSe-QDs (3.5 nm in diameter, 10^−6^ mol of particles) dispersed in hexane/toluene (ca. 10 ml) were added in the loaded reaction mixture, and the reaction flask was kept at 100~120 °C under vacuum for 30 min to remove the hexane/toluene and other undesired materials, such as moisture/air, of low vapor pressure. Afterward, the solution was heated to 230~240 °C under nitrogen flow where the shell growth was performed. As a first injection for shell growth, it was used only 1.1 ml of the Zn (0.1 M) and S (0.1 M) precursor solution. This is followed by alternating addition of Zn and S precursors, respectively to complete the CdSe cores with ZnS shell took about 5~6 h in total. The solution was then kept for overnight at 150~160 °C and finally cooled to room temperature. The synthesized QDs were purified with hexane/toluene and methanol extraction several times until the methanol phase was clear (the synthesis route is given briefly in Fig. 1).

**Figure 2:**
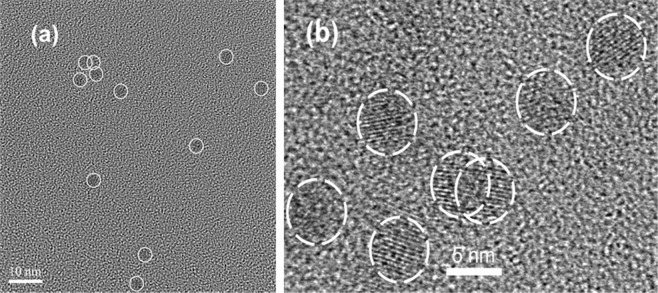
(**a**) HRTEM image of CdSe, average size 3.5 nm (scale bar 10 nm) (**b**) HRTEM image of CdSe/ZnS average size 5.2 nm (scale bar 5 nm).

**Figure 3:**
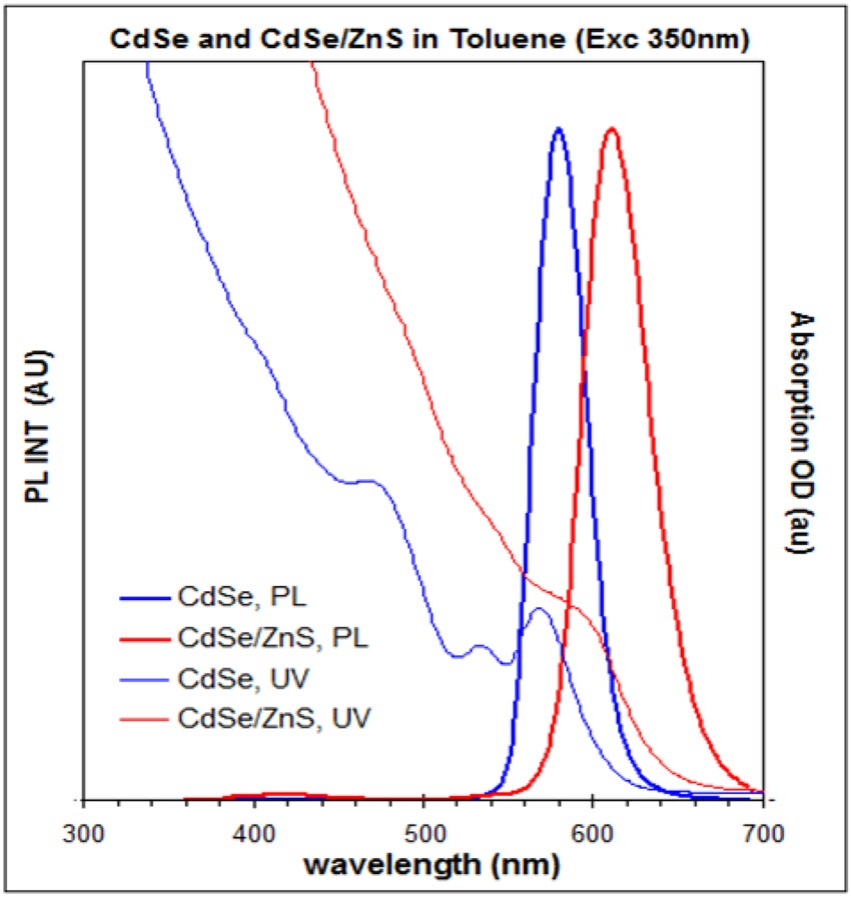
Absorption and Photoluminescence of CdSe (Blue colour QY = 34%) core and CdSe/ZnS (Red colour QY = 38%) core/shell spectra in toluene.

It is noteworthy to mention that we have added 5.5 multilayer (ML) of calculated materials and achieved ca. 3 ML ZnS shell based on our HRTEM results (Fig. 2(a),(b)). Figure 3 shows the optical properties of the resulting CdSe purified QDs in toluene. The CdSe QDs exhibiting UV absorption peaking at 568 nm (blue thin line, suggesting a diameter of ca 3.5 nm) and photoemission peaking at 582 nm (blue thick line, with excitation wavelength at 350 nm). The red spectra illustrated in Fig. 3 are the UV absorption and PL emission of core/shell QDs at 602 nm in toluene.

### Fabrication of working electrode with quantum dots

The working electrode of proposed 3-CP electrochemical was fabricated using chemical binder onto GCE. The GCE was coated with the ethanolic slurry of CdSe/ZnS QDs as uniform thin layer and kept in room condition to dehydrate the samples. To develop the binding strength between GCE and CdSe/ZnS QDs, a drop of 5% nafion suspended in ethanol was applied on dry modified GCE & introduced in an oven at 35 °C for short time (30 min) to dry the GCE completely. The proposed electrochemical sensor was attached with a Keithly electrometer, where CdSe/ZnS QD/binder/GCE was performed as working and Pt-wire as counter electrode respectively. 3-CP solution (between 1.0 mM and 0.1 nM) were prepared and used as target analytes. The calibration curve was plotted by using current versus concentration relation. By using the slope of calibration curve, the detection limit and sensitivity of 3-CP chemical sensor were calculated. Considering the regression co-efficient value (r^2^), the linear dynamic region from calibration curve was measured. It should be noticed that this electrochemical sensor is simple with two electrodes system. The phosphate buffer medium in detecting beaker was kept 10.0 mL as constant through the electrochemical experiments.

## Results and Discussions

The QDs were synthesized by using the formerly published works with the TOP, TOPO, HDA or SA^70,71^. Figure 3 shows the optical properties of the resulting CdSe purified QDs in toluene. The CdSe QDs are exhibiting UV absorption peaking at 568 nm (blue thin line, suggesting a diameter of ca 3.5 nm) and photoemission peaking at 582 nm (blue thick line, with excitation wavelength at 350 nm). The red spectra illustrated in Fig. 3 are the UV absorption and PL emission of core/shell QDs at 602 nm in toluene. Moreover, it was fabricated the ZnS shell around the CdSe cores by using the SILAR method. It is noteworthy to mention that we have added 5.5 ML of calculated materials and achieved ca. 3 ML ZnS shell based on our HRTEM results (Fig. 2(a),(b)).

### Detection of 3-CP by electrochemical approach

**Figure 4:**
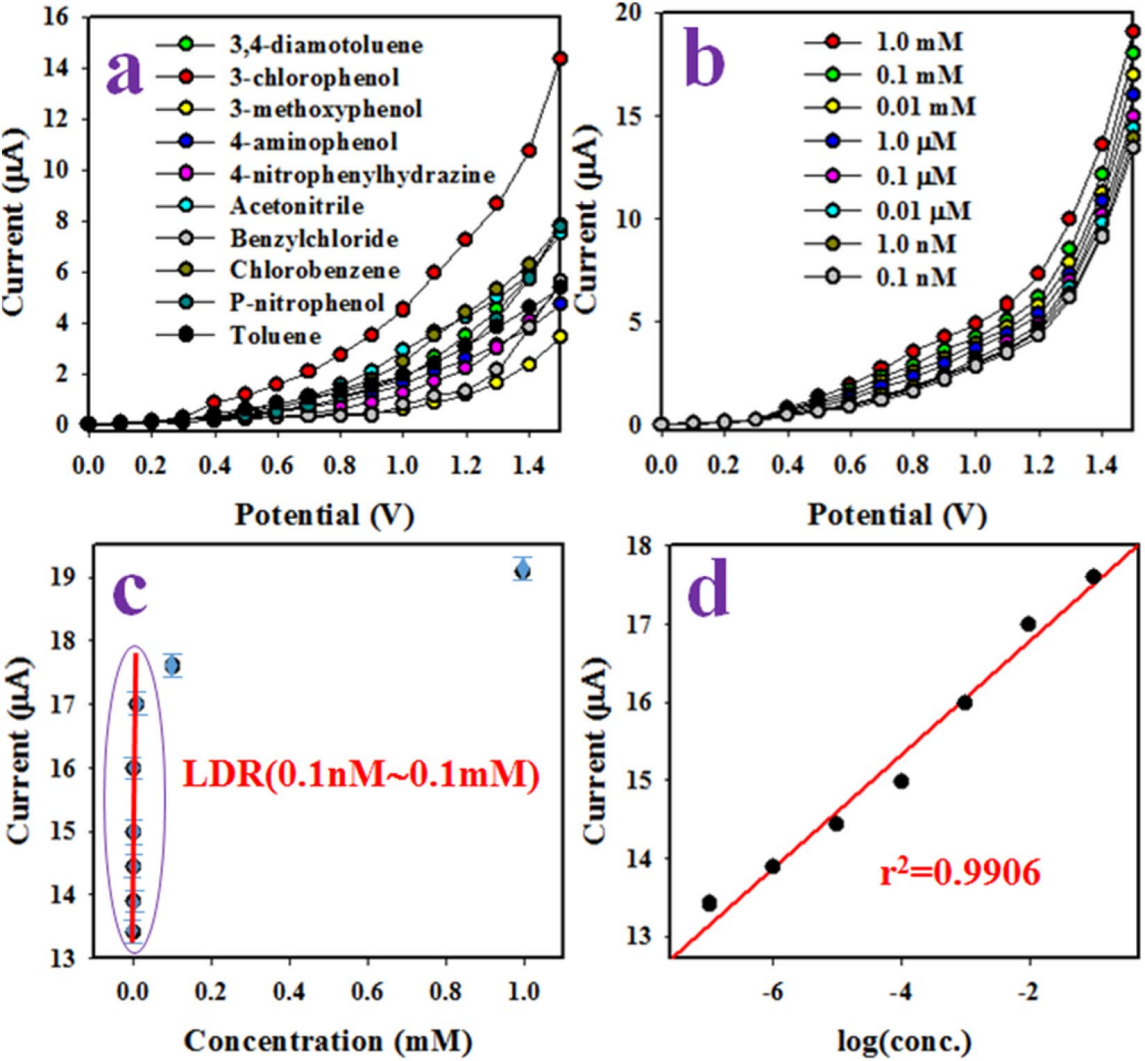
The optimization of 3-CP electrochemical sensor based on CdSe/ZnS QD/binder/GCE. (**a**) Selectivity (Contaminants have the identical concentration, 1.0 mM), (**b**) Electrochemical responses with concentration variation of 3-CP, (**c**) calibration plot, and (**d**) Evaluation of linearity (LRD).

The proposed application of 3-CP electrochemical sensor based on CdSe/ZnS QD/binder/GCE was to detect of 3-CP by electrochemical approach in phosphate buffer solution. The proposed sensor was developed by coating of GCE with ethanolic slurry of CdSe/ZnS QD as very thin uniform layer. As binding agent, it was used the 5% nafion suspended in the ethanol, which was enhanced the binding strength between GCE and CdSe/ZnS QDs. Thus, the applied nafion was improved the binding strength in addition to increase the electron transport rate of the proposed electro-chemical sensor^72–77^. To test the proposed selectivity of chemical sensor, the number of environmental contaminants with the concentration of 0.1 µM and applied potential ranging from 0 to +1.5 V were investigated in phosphate buffer solution at neutral pH. Figure 4(a) represents the electrochemical responses of 3,4-diaminotoluene, 3-chlorophenol, 3-methoxyphenol, 4-aminophenol, 4-nitrophenyhydrazine, acetonitrile, benzylchloride, chlorobenzene, p-nitrophenol, toluene, and among the toxins (identical concentration, 1.0 mM), 3-CP shows the highest electrochemical response. The electrochemical sensor based on CdSe/ZnS QD/binder/GCE was applied to analyze 3-CP with various concentration ranging from 1.0 mM to 0.1 nM as illustrated in Fig. 4(b). Figure 4(b) shows the electrochemical responses are distinguished from lower to higher 3-CP concentrations as expected. Therefore, this exploration is provided the evidence that the electrochemical responses are varied with 3-CP concentrations in phosphate buffer phase. To plot of calibration curve, the relation of current versus concentration of 3-CP was plotted. The current data was isolated from Fig. 4(b) at applied potential +1.5 V as demonstrated in Fig. 4(c).

The 3-CP based sensor’s sensitivity and detection limit were calculated by using the calibration curve. The obtained values are 3.6392 µA µM^−1^ cm^−2^ and 26.09 ± 1.30 pM respectively based on signal-to-noise ratio of 3. Obviously, the proposed sensitivity and detection limit (DL) are found quite satisfactory. As it is demonstrated in Fig. 4(d) of current vs. log(conc. of 3-CP), the current data is fitted with the regression co-efficient r^2^ = 0.9906 over the concentration range of 0.1 nM∼0.1 mM, which is identified as linear dynamic range (LDR). As it is shown in Fig. 4(c), the current data is continuously distributed along the linear plot over range of 0.1 nM∼0.1 mM, which is provided the information about the reliability of the method.

**Figure 5:**
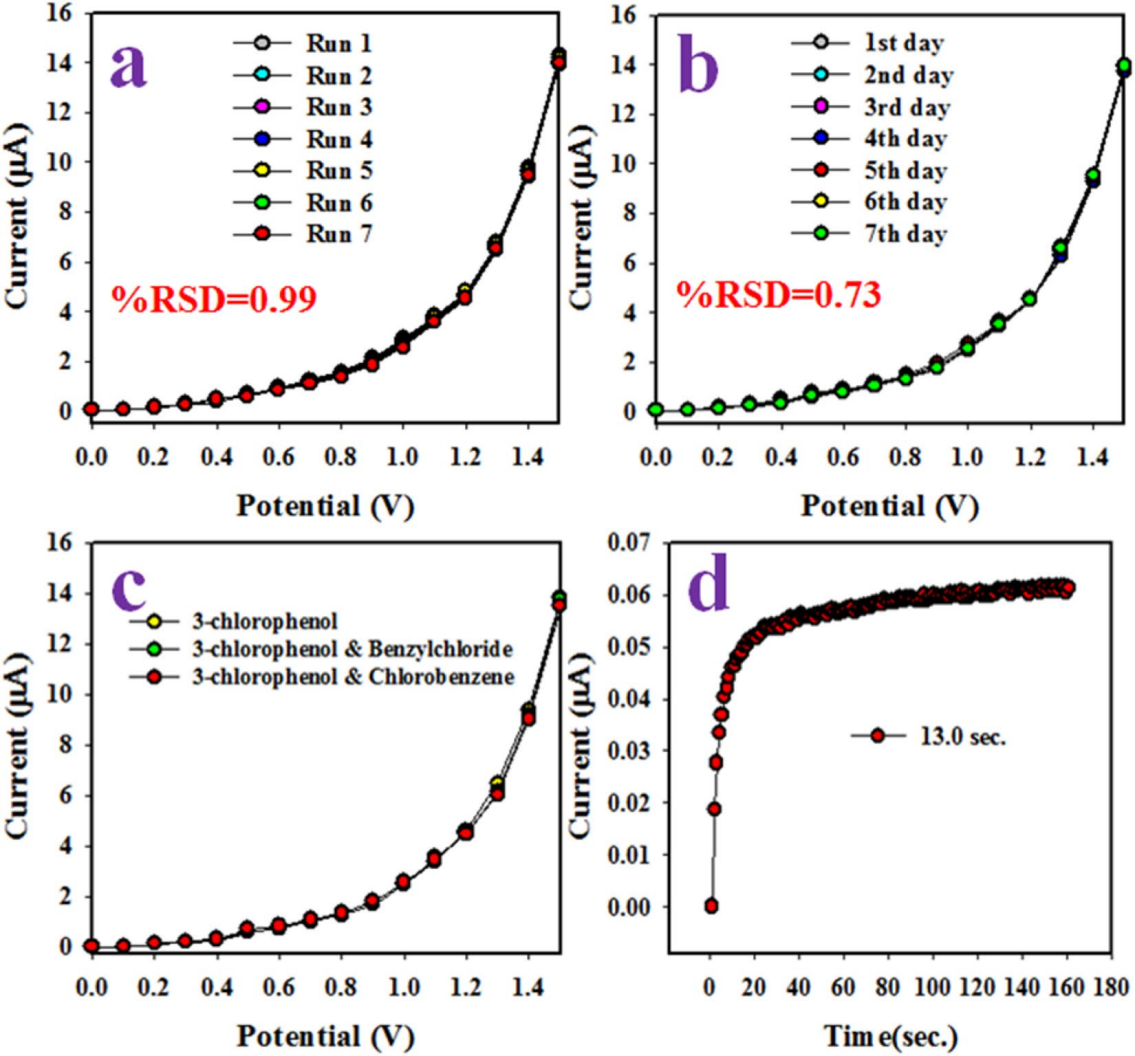
Optimization of sensor. (**a**) The reproducibility test of 3-CP chemical sensor based on CdSe/ZnS QD/binder/GCE, (**b**) validity performances of sensor around seven days, (**c**) interference effect for estimation of projected sensor and (**d**) evaluation of response time.

The reproducibility performance of the electrochemical sensor is referred to the features of reliability test. This test of 3-CP sensor was executed in 0.1 µM concentration and applied potential in 0∼ +1.5 V as represented in Fig. 5(a). As it is observed from Fig. 5(a), the seven runs are completely replicated and indistinguishable. The intensity of electrochemical responses is not altered even washing of fabricated electrode after each run. The percentage of relative standard deviation of current at applied potential +1.5 V was estimated and it is found to be 0.99%, which referred to highly precious. From this reproducibility performance, it can be summarized that the proposed 3-CP chemical sensor based on CdSe/ZnS QD/binder/GCE is able to detect the 3-CP in real environmental as well as extracted samples with high accuracy. The similar analogous test was done as illustrated in Fig. 5(b) for elongated period to measure the stability of proposed 3-CP sensor in phosphate buffer medium with desired outcome. As it is perceived from Fig. 5(b), the similar result as in Fig. 5(a) is obtained with precious outcome for around seven days. Thus the proposed 3-CP sensor has long-term stability in phosphate buffer medium with constancy in results. In Fig. 5(c), the interference effect of 3-CP sensor in presence of other toxins such as chlorobenzene and benzylchloride was explored. As outcome of this experimental test, no interference effect was perceived. The response time of an electrochemical sensor is measured as efficiency and responses of sensor. This test was evaluated at 0.1 µM concentration of 3-CP as demonstrated in Fig. 5(d). Fast response time around 13.0 sec is obtained.

**Figure 6:**
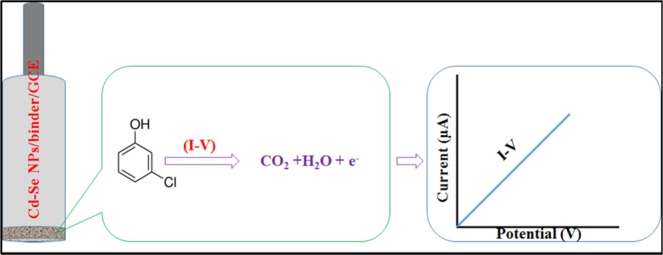
The electrochemical oxidation of 3-CP on CdSe/ZnS QD/binder/GCE matrix.

During the electrochemical oxidation of 3-CP based on CdSe/ZnS QD/binder/GCE sensor probe, initially 3-CP molecules are adsorbed onto the surface of working electrode (GCE). With the applied potential, 3-CP molecules are oxidized to carbon dioxide and water. In the same time, the electrons are generated and released onto CdSe/ZnS QD/binder/GCE surface, which are responsible to increase the conductivity of sensing probe in phosphate buffer medium (Fig. 6). As a result, the electrochemical responses are enhanced with increasing of concentration of 3-CP as illustrated in Fig. 4(b). The similar electrochemical oxidation phenomena of 3-CP have been reported elsewhere^62,78–81^.

**Figure 7:**
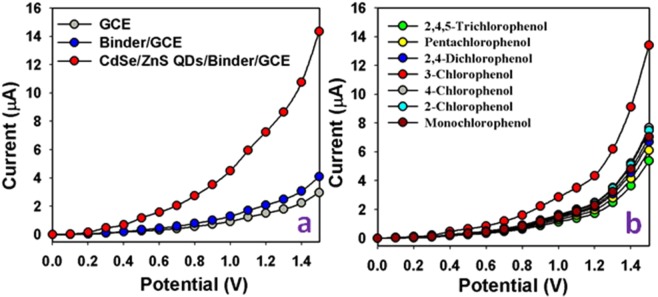
Optimization of Sensor probe. (**a**) Control experiment was performed with various modified GCE (Bare GCE, Binder/GCE and CdSe/ZnS QD/binder/GCE) in presence of target 3-CP in identical conditions (Analyte concentration is taken as 1.0 mM). (**b**) Electrochemical response with chlorophenol derivatives (1.0 mM) including target 3-CP.

A control experiment has been performed with various modified GCE (Bare GCE, Binder/GCE and CdSe/ZnS QD/binder/GCE) in the identical conditions in the presence of target 3-CP (Fig. 6a). It is observed that the highest current is exhibited with the fabricated CdSe/ZnS QD/binder/GCE sensor probe compared to bare and binder modified GCE electrodes. In presence of CdSe/ZnS QD material, 3-CP molecules are oxidized to carbon dioxide and water. So the electrons are generated and released, which are directly responsible to increase the conductivity of sensing probe. It has been performed an additional experiment with the derivatives of chlorophenol such as 4-chlorophenol, 2-chlorophenol, monochlorophenol, 2,4-dichlorophenol, pentachlorophenol, 2,4,5-trichlrophenol, including the target 3-chlorophenol. 3-CP is exhibited the highest electrochemical response compared to these derivatives, which is given in the Fig. 7b.

A comparison of related work based on DL and LDR are given in Table 1^82–86^. In a short, the 3-CP chemical sensor with CdSe/ZnS QD/binder/GCE matrix is showed satisfactory analytical performance such as good sensitivity (3.6392 µA µM^−1^ cm^−2^), wider LDR (0.1 nM∼0.1 mM) and very lower DL (26.09 ± 1.30 pM). It is also found as good reproducible response as well as reliable detection of 3-CP in environmental sample with quick response time.

**Table 1:** Comparison of sensor performances based on different modified electrodes by electrochemical approach for the detection of target 3-CP at room conditions.

Fabricated materials	DL	LDR	Ref
Poly (GMA-co-MTM)/PPy/CNT/HRP	0.44 µM	1.6–81.6 µM	^82^
CNT/PPy/HRP/ Au electrode	0.20 µM	1.6–12.8 µM	^83^
MPA–SAM/ Au electrode	0.51 µM	1–1000 µM	^84^
CD/GRs/CPE	0.09 µM	0.4–77 µM	^85^
Er2O3/CuO Nbs/GCE	0.09 nM	0.1 nM–10.0 mM	^86^
Cd-Se/ZnS QDs/Binder/GCE	26.09 pM	0.1 nM-0.1 mM	This work

*DL(Detection limit), LDR(Linear dynamic range), nM(Nanomole), µM(Micromole), pM (Picomole),

### Analysis of real environmental and extracted samples

For the validation, the CdSe/ZnS QD/binder/GCE sensor probe was implemented to detect 3-CP in real environmental samples by using recovery method. The real samples were collected from industrial waste effluent, extracted from PC-baby bottle, PC-water bottle, and PVC-food packaging bags. The resulted analysis data are summarized in Table 2 and it is found to be as quite accepted and satisfactory results.

**Table 2:** The analysis of real environmental and extracted samples by using CdSe/ZnS QD/binder/GCE sensor probe by recovery method.

Sample	Concentration added of 3-CP(µM)	Measured 3-CP conc.^a^ by CdSe/ZnS QD/binder/GCE (µM)			Average recovery^b^ (%)	RSD^c^ (%) (n = 3)
		R1	R2	R3		
Industrial contaminants	0.01000	0.01039	0.01028	0.01016	102.77	1.12
PC- baby bottle	0.01000	0.01002	0.00994	0.01014	100.33	1.00
PC- water bottle	0.01000	0.01013	0.01008	0.01013	101.13	0.29
PVC- food packaging bag	0.01000	0.00979	0.00996	0.01006	99.38	1.37

^a^Mean of three repeated determination (signal to noise ratio 3) CdSe/ZnS QD/binder/GCE.

^b^Concentration of 3-CP determined/Concentration taken. (Unit: µM)

^c^Relative standard deviation value indicates precision among three repeated measurements (R1, R2, R3).

## Conclusion

In this work, photolumenescent CdSe/ZnS QDs have been prepared and significantly utilized for efficient selective electrochemical sensor applications. The proposed selective 3-CP electrochemical sensor was fabricated by using CdSe/ZnS QD deposited onto flat GCE with conducting 5% nafion binder and employed to detect the target 3-CP in phosphate buffer phase. For estimating the analytical performance, a calibration curve like concentration versus current of 3-CP was plotted. Then the slope of this resultant calibration curve was employed to determine the sensitivity (3.6392 µA µM^−1^ cm^−2^), LDR (0.1 nM∼0.1 mM), and DL (26.09 ± 1.30 pM). The proposed 3-CP sensor was successfully implemented to execute the reproducibility test and analyze the real environmental samples for the confirmation. It is introduced a new route for the development of selective and sensitive chemical sensor to detect the unsafe pollutant existed in the environment by using core-shell quantum dots coated GCE by electrochemical approach for the safety of environmental and healthcare fields in broad scales.
